# Effectiveness and Validation of the Italian Translation of the Low Anterior Resection Syndrome Score in an Italian High-Volume University Hospital

**DOI:** 10.3389/fsurg.2022.917224

**Published:** 2022-06-20

**Authors:** Veronica De Simone, Francesco Litta, Roberto Persiani, Gianluca Rizzo, Luigi Sofo, Roberta Menghi, Francesco Santullo, Alberto Biondi, Claudio Coco, Franco Sacchetti, Fabio Longo, Miriam Attalla El Halabieh, Rossana Moroni, Carlo Ratto

**Affiliations:** ^1^ Proctology Unit, Fondazione Policlinico Universitario “A. Gemelli” IRCCS, Rome, Italy; ^2^ General Surgery Unit, Fondazione Policlinico Universitario “A. Gemelli” IRCCS, Rome, Italy; ^3^ General Surgery II Unit, Fondazione Policlinico Universitario “A. Gemelli” IRCCS, Rome, Italy; ^4^ Abdominal Surgery Unit, Fondazione Policlinico Universitario “A. Gemelli” IRCCS, Rome, Italy; ^5^ Digestive Surgery Unit, Fondazione Policlinico Universitario “A. Gemelli” IRCCS, Rome, Italy; ^6^ Peritoneal and Retroperitoneal Surgery Unit, Fondazione Policlinico Universitario “A. Gemelli” IRCCS, Rome, Italy; ^7^ Scientific Direction, Fondazione Policlinico Universitario “A. Gemelli” IRCCS, Rome, Italy

**Keywords:** rectal cancer, low anterior resection, low anterior resection syndrome, quality of life, functional outcomes

## Abstract

**Background:**

The low anterior resection syndrome (LARS) score is a validated questionnaire developed in Denmark to measure the severity of bowel dysfunction after low anterior resection. This retrospective study aimed to assess the effectiveness of the LARS score in the Italian language in a population of Italian patients who underwent low anterior resection for rectal cancer. The convergent and discriminative validity and the test-retest reliability of the score were investigated.

**Methods:**

A cohort of two hundred and five patients treated with low anterior resection were enrolled in an Italian high-volume university hospital between January 2000 and April 2018. The Italian version of the LARS score (tested twice), as translated from English original version, a single question on quality of life and the EORTC QLQ-C30 questionnaire were submitted to patients.

**Results:**

A high proportion of patients showed a perfect or moderate fit between the LARS score and QoL categories (convergent validity, *p* < 0.0005). All differences regarding the items of the European Organization for Research and Treatment of Cancer Quality of Life Questionnaire – Core 30 (EORTC QLQ-C30) functional scales were statistically significant (*p* < 0.0005). The LARS score was able to discriminate between groups of patients who received or did not receive preoperative chemoradiotherapy (*p* < 0.0005) and those who received total or partial mesorectal excision (*p* < 0.0005). The test-retest reliability was excellent (intraclass correlation coefficient 0.96).

**Conclusion:**

The Italian translation of the LARS score is an easy and reliable tool for assessing bowel dysfunction after low anterior resection and its routine use in clinical practice should be recommended.

**Trial registration number** at www.clinicaltrials.gov: NCT04406311.

## Introduction

Colorectal cancer represents the third most common neoplasm in men (12.0%) and the second in women (11.2%) in Italy, with 43,700 new diagnoses expected in 2020 (23,400 in men and 20,300 in women) (1). The rectum is the most frequently involved site among colorectal tumours (approximately 35% of cases).

Increasing attention has been recently paid to the outcomes of surgical treatment in terms of patient anorectal function and quality of life (QoL). Currently, the majority of patients affected by rectal carcinoma undergo a sphincter-sparing procedure, avoiding a permanent colostomy.

Up to 80% of patients undergoing low anterior resection (LAR) will have at least some degree of bowel dysfunction (2–4); for this reason, the term low anterior resection syndrome (LARS) has been coined to describe this complex functional condition (3). The main symptoms included in this syndrome are as follows: incontinence of gas and/or liquid or solid stools, constipation, urgency, fragmentation and frequent bowel movements. In addition, a worsening of QoL has been observed in patients with severe LARS symptoms (5).

Due to the importance and high prevalence of this condition, the so-called LARS score has been introduced (6) to identify a reliable tool for assessing severity and determining the type of treatment (7). The score has been validated in several languages, including English (8), Chinese (9), Lithuanian (10), Swedish, Spanish, German, Danish (in a consolidated international validation) (11), Dutch (12) and many others (13, 14).

The primary aim of this study was to assess the effectiveness of the LARS score in the Italian language in a population of Italian patients who underwent LAR for rectal cancer. Moreover, the study provided the opportunity to investigate convergent and discriminatory validity and to retest the reliability of the score.

## Methods

This retrospective, observational study included rectal cancer patients treated by LAR with total mesorectal excision (TME) or partial mesorectal excision (PME) between January 2000 and April 2018. The study was reported according to the Strengthening the Reporting of Observational Studies in Epidemiology (STROBE) statement for cohort studies (15) and was approved by the local Ethical Committee (Protocol ID 3358). The present study was registered at www.clinicaltrials.gov (NCT04406311) on May 2020, when a validated Italian translation of the LARS was not yet available. All patients provided written informed consent.

### Translation

**Figure 1:**
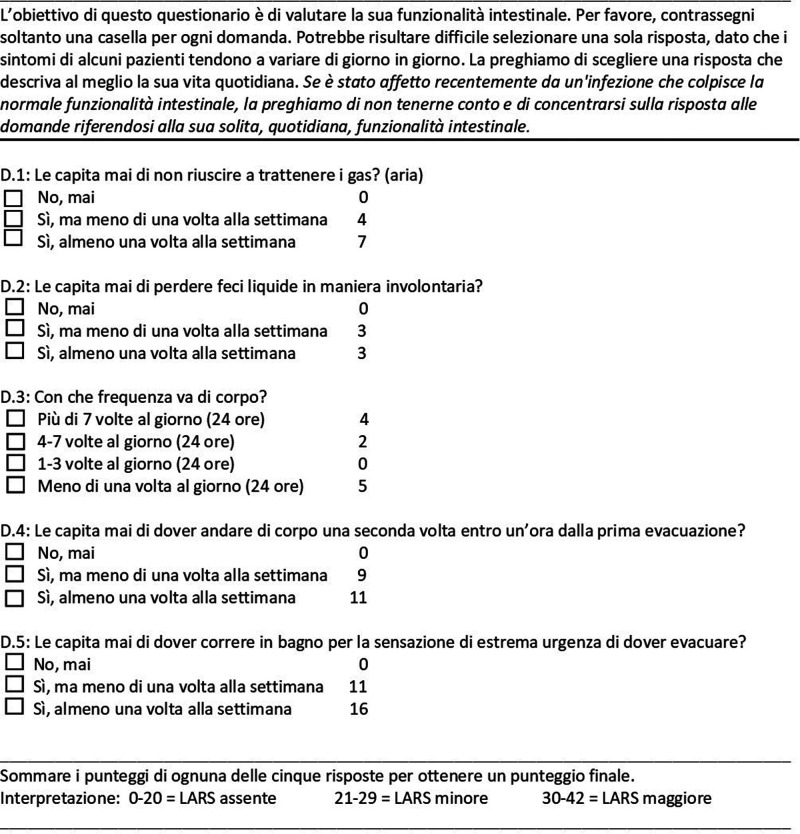
The Italian version of the low anterior resection syndrome (LARS) score questionnaire.

The validated English version of the LARS questionnaire was translated into the Italian language. The translation was performed by two independent professional translators. The translators discussed any discrepancies between their translations until an agreed-upon version was reached. A third native English translator translated the Italian version into English. Subsequently, the two English versions (the initial version and the new version) were compared, and the final version in Italian was elaborated (Figure 1).

### Data Collection and Participants

Six surgical units of the “Fondazione Policlinico Universitario A. Gemelli, IRCCS” of Rome participated in the data collection. The inclusion criteria were as follows: diagnosis of rectal cancer (between 0 and 15 cm from the anal verge); treatment with anterior rectal resection surgery (open, laparoscopic, robotic or transanal approach) with total or partial mesorectal excision (TME or PME); if a stoma has been created, intestinal continuity must have been restored for at least 24 months (by April 2018). The exclusion criteria were: dementia; metastatic or recurrent disease; other intestinal diseases (including Crohn’s disease, ulcerative colitis); patients with a stoma or with intestinal continuity restored for less than 24 months; and patients with problems understanding the Italian language. Eligible patients received an invitation to complete the European Organization for Research and Treatment of Cancer Quality of Life Questionnaire – Core 30 (EORTC QLQ-C30), two copies of the LARS questionnaire (administered 1–2 weeks apart), and a single question about QoL, which was added for validation purposes.

Each surgical unit was responsible for the truthfulness of the data collected and provided.

### Patient-Reported Outcome Measures

#### Lars Score

The LARS questionnaire translated into Italian was administered to all patients enrolled in the study. The LARS score was originally developed in Denmark with a population of rectal cancer patients. The score is based on five questions regarding bowel dysfunction that were selected from 26 candidate items on the basis of their high correlation with patient-reported QoL. The scores of the five subscales are summed to produce a total score ranging from 0 to 42 points. Patients were classified into three groups according to their total score: 0–20 points: no LARS; 21–29 points: minor LARS; and 30–42 points: major LARS (6).

#### Single Question on Qol

A single question on QoL was added to the LARS score to investigate convergent validity. The question, “Complessivamente, in che modo la sua funzione intestinale influisce sulla sua qualità della vita?” (in English, “Overall, how does bowel function affect your quality of life?”), was answered with one of the following options: “per niente”, “un po’”, “parecchio”, “moltissimo” (in English, “not at all”, “a little”, “quite a bit”, “a lot”). This question was previously used for the development and validation of the LARS score in other countries (6, 11, 12). To evaluate the degree of agreement between the 3 LARS score categories and the single QoL question, the last question was grouped as follows: “not at all” = no impact on QoL; “a little” = minor impact on QoL; “quite a bit” + “a lot” = major impact.

#### EORTC QLQ-C30

The EORTC QLQ-C30 questionnaire (16, 17) is a validated and specific tool for evaluating the QoL of cancer patients. It consists of 30 questions that provide a global QoL scale, five functional scales (i.e., physical, role-playing, emotional, cognitive, social), three symptom scales (fatigue, nausea and vomiting, pain) and six individual factors (dyspnoea, insomnia, loss of appetite, constipation, diarrhoea, financial difficulties). The scores for each scale are combined to produce a score ranging from 0 to 100. For the purpose of this study, only the functional scales and the global QoL scale were used. A high score on a functional scale represented a good level of function.

### Statistical Analysis

Based on previous validation studies conducted in other countries (8–12), it was determined that the sample should include at least 200 patients. The clinical and demographic features of the sample are described using descriptive statistics. Quantitative variables are described using the following measures: mean and standard deviation. Qualitative variables are summarized as absolute and percentage frequencies.

#### Convergent Validity

The LARS score data are presented as the median and interquartile range (IQR). Based on the responses to the single QoL question, the patients were grouped into three categories: no impact, minor impact or some/major impact of bowel function on QoL. The fit between the QoL category and LARS score category was investigated and was considered perfect when patients reported no LARS and no impact on QoL, minor LARS and a minor impact on QoL, or major LARS and some/major impact on QoL. A box and whisker plot analysis was used to illustrate the differences in the numerical LARS score among QoL categories, and any difference was tested by the Kruskal-Wallis test. Convergent validity was explored by investigating the association between the LARS categories and the five functional subscales and the global QoL scale of the EORTC QLQ-C30. EORTC QLQ-C30 scores were calculated. The Kruskal-Wallis test was used to perform all comparisons.

#### Discriminative Validity

The ability of the LARS score to differentiate among groups of patients was evaluated with the Mann-Whitney test. Similar to previous validation studies (6, 8, 11, 12), the clinically relevant subgroups were based on preoperative chemoradiotherapy (CRT), type of surgery (TME/PME), and age (cut-off of 69 years).

#### Test-Retest Reliability

Test-retest reliability is a key aspect of all health measures (18). To examine the test-retest reliability of the LARS score, all patients were sent a second LARS questionnaire 1–2 weeks after they completed the first one, and they all were asked to complete the questionnaire again. Agreement between tests for each of the five LARS score items and for the LARS score classification is presented as the proportion with 95% CI. A Bland-Altman plot with 95% limits of agreement is also presented, as is the intraclass correlation coefficient (ICC). An ICC above 80 is considered excellent agreement. A p-value less than 0.05 was considered statistically significant.

All statistical analyses were performed using SPSS® version 25.0 for Windows® software (SPSS, Chicago, IL, USA).

## Results

Two hundred five patients (117 males, 88 females; mean age 67.7 ± 11.0 years) were enrolled in the study and returned a completed LARS score questionnaire. Only 42.0% of the respondents underwent preoperative CRT, and 77.6% of them had undergone TME. 53.2% of the patients underwent a laparoscopic approach; the others 18.6%, 14.1% and 14.1% underwent an open, robotic and transanal approach respectively. According to the LARS score, 74 (36.1%) patients had major LARS, 55 (26.8%) had minor LARS, and 76 (37.1%) had no LARS. A detailed description of the patients’ characteristics is provided in Table 1. Seventy-two patients (35.1%) were followed up in the outpatient clinic, 66 patients (32.2%) were followed up by e-mail, and 67 (32.7%) completed a telephone interview.

**Table 1:** Baseline characteristics of patients (*N* = 205).

Gender (*n*, %)		
Males	117	57.1
Females	88	42.9
Age (mean, SD)	67.7	11
Distance of the cancer from the anal verge (cm) (mean, SD)	8.95	4
Distance of the anastomosis from the anal verge (cm) (mean, SD)	4.72	3
Neoadjuvant radiotheraphy (*n*, %)		
NO	119	58.0
YES	86	42.0
Resection type (*n*, %)		
TME	159	77.6
PME	46	22.4
Surgical Approach (*n*, %)		
OPEN	38	18.6
LPS	109	53.2
ROBOTIC	29	14.1
TaTME	29	14.1
Stoma creation (*n*, %)		
NO	81	39.5
YES	123	60.0
LARS SCORE AT QUESTIONNAIRE #1 (median, IQR)	27	19
LARS SCORE CLASSES #1 (*n*, %)		
No LARS	76	37.1
Minor LARS	55	26.8
Major LARS	74	36.1
LARS SCORE AT QUESTIONNAIRE #2 (median, IQR)	25.5	19
LARS SCORE CLASSES #2 (*n*, %)		
No LARS	76	37.1
Minor LARS	54	26.3
Major LARS	74	36.1
No response	1	0.5
QOL SINGOLA (*n*, %)		
Not at all	55	26.8
Very little	50	24.4
Somewhat	74	36.1
A lot	26	12.7

*Abbreviations: TME, Total mesorectal excision; PME, partial mesorectal excision; LARS, low anterior resection syndrome; LPS, laparoscopic; TaTME, transanal total mesorectal excision.*

### Convergent Validity

**Figure 2:**
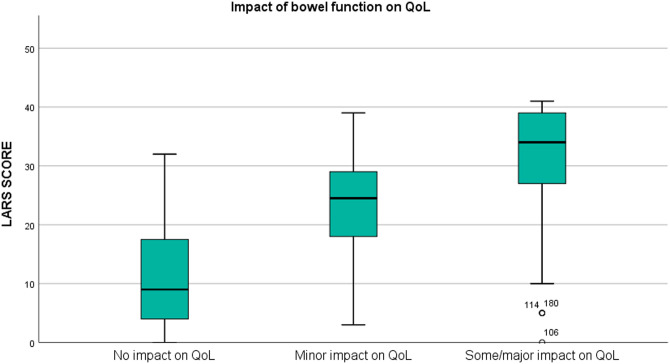
Box plot illustrating the association between the LARS score and the impact of bowel function on quality of life (QoL) (*p* < 0.0005).

The proportion of patients with a perfect fit between the QoL category and the LARS score category was 64.3%; a moderate fit was found for 29.8%, and no fit was found for 5.9% (Table 2). For respondents who reported that bowel problems had no impact on QoL (*n* = 55), the median (IQR) LARS score was 9 (4–18), whereas for those who reported that it had a minor impact on QoL (*n* = 50), the median (IQR) LARS score was 24.5 (17.5–29). Patients who reported that bowel problems had some/a major impact on QoL (*n* = 100) had a median (IQR) LARS score of 34 (27–39). Differences in the LARS score among QoL categories were highly significant (*p* < 0.0005) (Figure 2). The three LARS categories were also compared with the EORTC QLQ-C30 functional scales (Physical functioning, Emotional functioning, Role functioning, Cognitive functioning, Social functioning) and the global health score. Table 3 presents the main results of these comparisons; all differences regarding all items of the EORTC QLQ-C30 functional scales were statistically significant.

**Table 2:** Fit between LARS category and QoL category.

	No impact on QoL	Minor impact on QoL	Major impat on QoL
No LARS	46 (22.4%)	20 (9.8%)	10 (4.9%)
Minor LARS	7 (3.4%)	22 (10.7%)	26 (12.7%)
Major LARS	2 (1.0%)	8 (3.9%)	64 (31.2%)

*Perfect fit: 64.3%.*

*Moderate fit: 29.8%.*

*No fit: 5.9%.*

**Table 3:** Median score, 1st and 3rd quartile of the functional scales compared between the LARS categories.

	No LARS			Minor LARS			Major LARS			*p*-value
	median	1st quartile	3rd quartile	median	1st quartile	3rd quartile	median	1st quartile	3rd quartile	
GHS	833	750	100	750	667	833	667	500	750	<0.0005
PHYS_FUNCT_SCORE	100	80	100	93	80	100	87	67	93	<0.0005
EMOT_FUNCT_SCORE	100	83	100	100	83	100	83	67	94	<0.0005
ROLE_FUNCT_SCORE	100	100	100	100	67	100	75	67	100	<0.0005
COGN_FUNCT_SCORE	100	100	100	100	83	100	83	67	100	<0.0005
SOCIAL_SCORE	100	100	100	83	67	100	67	50	100	<0.0005

### Discriminative Validity

**Figure 3:**
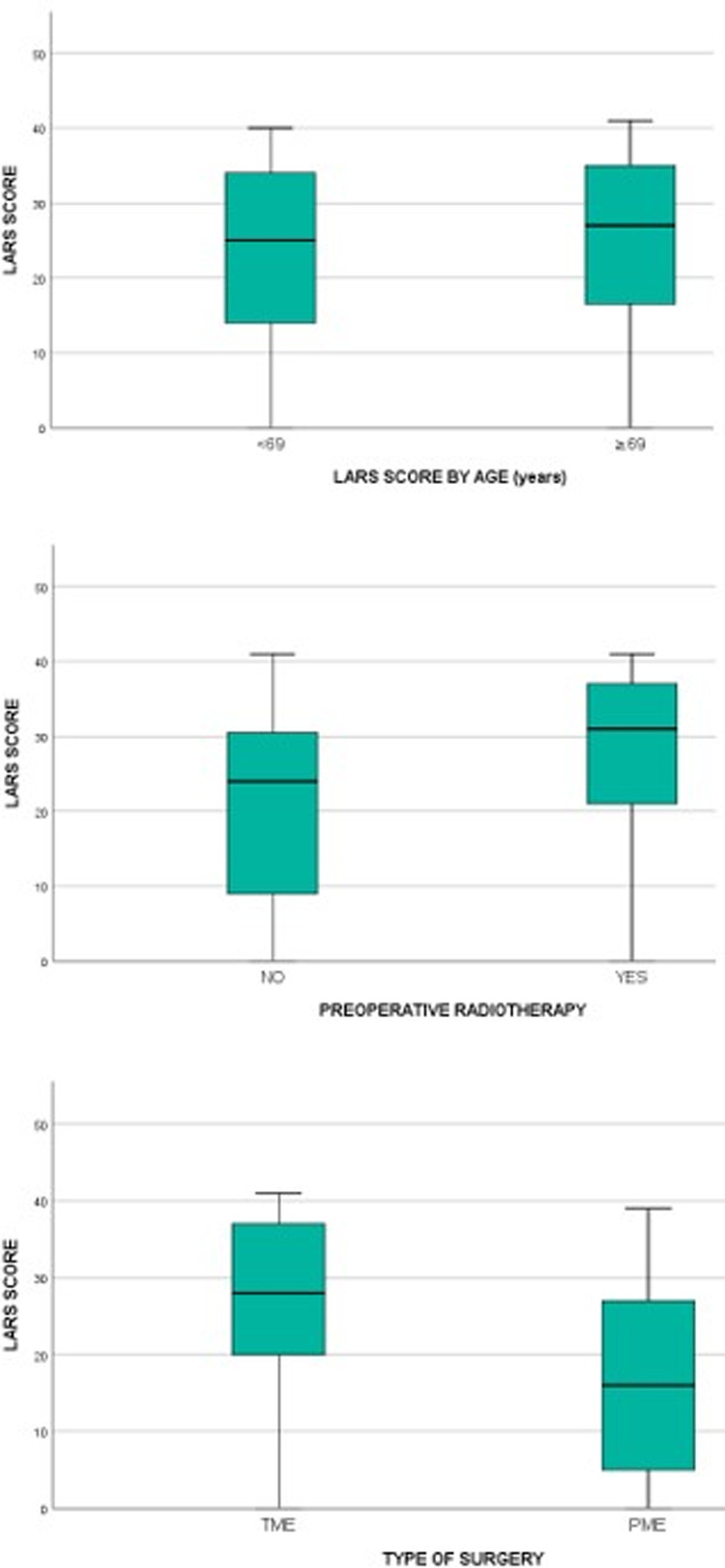
Comparison of LARS scores in groups of patients that differ by age (*p* = 0.534), preoperative chemoradiotherapy (*p* < 0.0005) and type of surgery (*p* < 0.0005).

As shown in Figure 3, the LARS scores of patients who underwent preoperative CRT (*n* = 86; median  = 31, IQR = 21–37) were significantly higher than those of patients who proceeded directly to surgery (*n* = 119; median = 24, IQR = 9–31) (*p* < 0.0005). The LARS score was also able to discriminate between PME patients (*n* = 46; median = 16, IQR = 5–27.5) and TME patients (*n* = 159; median = 28, IQR = 20–37) (*p* < 0.0005). The LARS score was not able to discriminate between <69-year-old patients and ≥69-year-old patients (*p* = 0.534).

### Reliability

**Figure 4:**
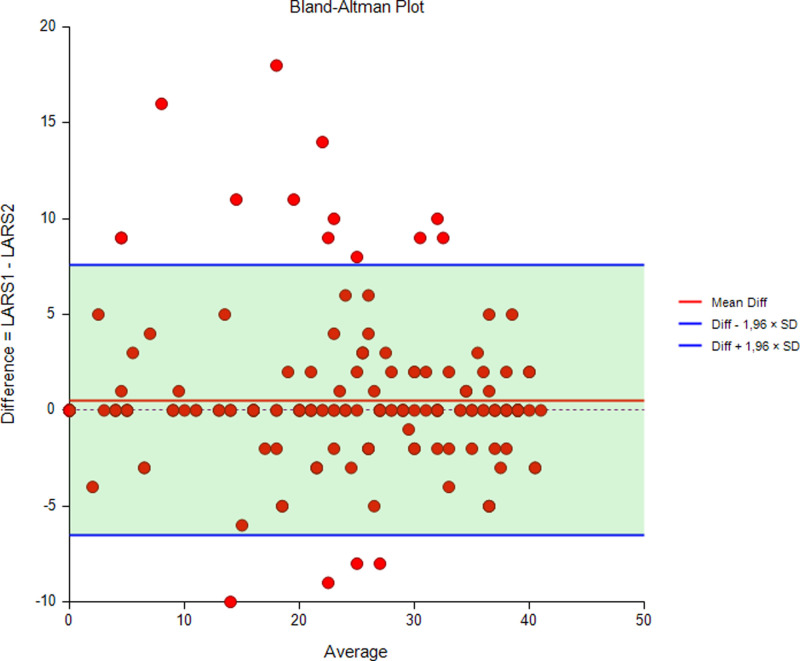
Bland-Altman plot with 95% limits of agreement illustrating the difference between LARS scores at the first and second tests.

All 205 patients were asked to complete the LARS score twice, and 204 responded to both questionnaires (response rate 99%). The median (IQR) number of days between tests was 11 (9–16). The Bland-Altman plot with 95% limits of agreement (−6.5 to 7.5) in Figure 4 illustrates the difference between the LARS scores on the first and second tests. This difference was statistically significant (*p* = 0.046).

The degree of agreement between the initial test and the retest for each of the LARS categories (no, minor, major LARS) is presented in Table 4. The results showed that 88.7% of the patients remained in the same LARS category at both tests, 11.2% differed by one category and no one differed by two categories between tests. The ICC was 0.96, indicating excellent reliability.

**Table 4:** Agreement between first and second LARS score category.

		LARS 2 CATEGORY		
		No impact	Minor impact	Major impact
LARS 1 CATEGORY	No impact	34.3%	2.9%	0.0%
	Minor impact	2.9%	21.1%	2.9%
	Major impact	0.0%	2.5%	33.3%

*Perfect fit: 88.7%.*

*Moderate fit: 11.2%.*

*No fit: 0.0%.*

## Discussion

This study demonstrated the effectiveness of the Italian translation of the LARS score in a cohort of Italian patients with rectal cancer, with a strong association between the LARS score and QoL. As regard as the validity of the score, the present version of the LARS score allowed us to discriminate between the different kinds of mesorectal resection (TME vs. PME) and patients who did and did not receive neoadjuvant CRT (19). The LARS score could not discriminate between patients younger than 69 years old and those aged 69 years and older. Moreover, the test-retest reliability was high. Table 5 compares the data reported in the previous validation studies of the LARS score in different populations to the Italian results.

**Table 5:** Comparison between different studies aimed to validate the LARS score (values expressed in %).

	Lars categories			Convergent validity			Discriminative validity			TME/PME	RT/ no RT	Reliability ICC
	No LARS	Minor LARS	Major LARS	Perfect fit	Moderate fit	No fit	Age groups	TME/PME	RT/no RT			
DANISH	35.4	24.9	39.7	62.2	31.9	5.9	–	yes	yes	60/40	21/79	0.46 to 0.95^a^
ENGLISH	29.7	22.8	47.5	51.5	44.1	4.5	yes	yes	yes	81/19	31/69	0.83
INTERNATIONAL^b^	28.1	19.5	52.4	60.7	34.2	5.1	yes	yes	yes	75/25	55/45	0.91
CHINESE	23.5	21.6	54.9	78.0	18.0	4.0	no	–	yes	–	28/74	0.86^c^
DUTCH	21.8	18.8	59.4	41.8	49.7	8.5	yes	yes	yes	82/18	90/10	0.79
LITHUANIAN	56.0	24.0	25.0	54.5	38.0	7.5	no	–	no	–	49/51	0.92
ITALIAN	37.1	26.3	36.1	64.3	29.8	5.9	no	yes	yes	77/23	42/58	0.96

*Abbreviations: TME, total mesorectal excision; PME, partial mesorectal excision; RT, radiotherapy; ICC, intraclass correlation coefficient.*

^a^ *Kappa values.*

^b^ *median value of the four Countries included.*

^c^ *Spearman correlation coefficient.*

Our results were consistent with previous reports (8, 11, 12), showing a higher proportion of major LARS after TME than after PME. Indeed, in the Italian population with rectal cancer, 46% of patients complained of major LARS after TME. In earlier validation studies (8–11), 47–59% of patients reported major LARS after TME, while a higher percentage of major LARS (59.4%) was recorded in the Dutch group (12). The wide difference in the percentage of patients who had neoadjuvant CRT could explain the variable distribution of major LARS among different countries (in the Dutch population, 90% of patients received neoadjuvant CRT; in Italy, 42% did). In accordance with other validation results (8–12), patients treated with preoperative CRT had a significantly higher LARS score, confirming the negative impact of CRT on patient-reported QoL (19, 20).

In contrast to the Dutch and international validation (11, 12), no differences were found between age groups, as previously reported by Chinese and Lithuanian authors (9, 10). However, a larger sample size could have improved the discriminatory ability. As in previous validations (8, 11, 12), a single QoL category question was used to test convergent validity. The Italian results of perfect (64.3%), moderate (29.8%) and no fit (5.9%) were similar to those reported in the international validation (11).

To further investigate convergent validity, the EORTC QLQ-C30 functional and global scales were compared to the LARS score categories. There was a significant correlation between a higher LARS score and a worse QoL. As reported in the English validation (8), there was an association between the LARS scores and all the EORTC QLQ-C30 subscales, including the cognitive functioning subscale. When compared with English and other international validation studies (8, 11), the reliability of the LARS score was excellent. There was remarkable patient compliance with completion of the LARS score questionnaire, thus demonstrating that the LARS score is easy to understand and complete.

Recently, Resendiz and colleagues (21) published a case series of 147 patients from 3 referral centers, across a 4-year period, with the aim of validating the Italian version of the LARS score. In this context, the major strenght of our study was to consider a higher volume of patients coming from the same center allowing a homogeneity of the data. Moreover, considering a period of almost 20 years, in which there has been a clear technological evolution involving rectal cancer surgery, we believe we have given the idea of a greater applicability of the LARS score whatever the chosen approach (open, laparoscopic, robotic, transanal). Lastly, we compared and critically analyzed the Italian version with other validated scores.

This study has some limitations. It was performed at a single institution that is an Italian referral centre for rectal cancer, and the expertise of the surgeons involved and the high volume of patients treated can explain the favourable distribution of LARS score categories, including a lower percentage of major LARS, compared to similar validation studies. Moreover, since the primary objective of this study was to validate the Italian version of the LARS score, anorectal function was not homogeneously assessed before surgery. As reported in the previous validations the type of anastomosis performed (stapled or hand-sewn) was not considered as discriminatory outcome. The epidemiology of LARS in the rectal cancer population and the investigation of risk factors were not aims of this study. In the test-retest analysis, there was a short interval between tests because it was assumed that over a longer period, a change in bowel function could occur. However, a potential disadvantage of a short interval is an increased risk of patients copying their first questionnaire responses when answering the second questionnaire.

## Conclusion

The Italian translation of the LARS score is a valid tool for the assessment of bowel dysfunction after rectal cancer surgery in the Italian population. It has demonstrated a strong association with QoL and high convergent and discriminative validity and reliability comparable to earlier validations. The Italian version of the questionnaire is reliable, easy to understand and complete, and its routine use should be included in clinical practice.

## Data Availability

The raw data supporting the conclusions of this article will be made available by the authors, without undue reservation.
